# Direct detection of SARS-CoV-2 antisense and sense genomic RNA in human saliva by semi-autonomous fluorescence *in situ* hybridization: A proxy for contagiousness?

**DOI:** 10.1371/journal.pone.0256378

**Published:** 2021-08-17

**Authors:** Gijsbert J. Jansen, Marit Wiersma, Willem J. B. van Wamel, Inge D. Wijnberg

**Affiliations:** 1 Biotrack, Leeuwarden, The Netherlands; 2 Department of Medical Microbiology and Infectious Diseases, Erasmus Medical Center, Rotterdam, The Netherlands; 3 Coordination Centre for Expertise on Working Conditions & Health, Cluster Infectious Diseases and (micro)biology, Ministry of Defense, Doorn, The Netherlands; University of Helsinki: Helsingin Yliopisto, FINLAND

## Abstract

Saliva is a matrix which may act as a vector for pathogen transmission and may serve as a possible proxy for SARS-CoV-2 contagiousness. Therefore, the possibility of detection of intracellular SARS-CoV-2 in saliva by means of fluorescence *in situ* hybridization is tested, utilizing probes targeting the antisense or sense genomic RNA of SARS-CoV-2. This method was applied in a pilot study with saliva samples collected from healthy persons and those presenting with mild or moderate COVID-19 symptoms. In all participants, saliva appeared a suitable matrix for the detection of SARS-CoV-2. Among the healthy, mild COVID-19-symptomatic and moderate COVID-19-symptomatic persons, 0%, 90% and 100% tested positive for SARS-CoV-2, respectively. Moreover, the procedure allows for simultaneous measurement of viral load (‘presence’, sense genomic SARS-CoV-2 RNA) and viral replication (‘activity’, antisense genomic SARS-CoV-2 RNA) and may yield qualitative results. In addition, the visualization of DNA in the cells in saliva provides an additional cytological context to the validity and interpretability of the test results. The method described in this pilot study may be a valuable diagnostic tool for detection of SARS-CoV-2, distinguishing between ‘presence’ (viral load) and ‘activity’ (viral replication) of the virus. Moreover, the method potentially gives more information about possible contagiousness.

## Introduction

The rapid spread of a severe acute respiratory syndrome, caused by the Severe Acute Respiratory Syndrome Coronavirus-2 (SARS-CoV-2), has led to the global coronavirus disease of 2019 (COVID-19) pandemic. This development has had a major impact on diverse aspects, e.g. health(care), economy, and geo-political relations [1]. Despite the unprecedented speed at which SARS-CoV-2-specific vaccines are currently being developed and distributed, the key factor for controlling this pandemic and for measuring vaccine effectiveness in populations around the globe remains the adequate testing for the presence of the pathogen. As far as testing is concerned, a large scientific and non-scientifically validated bibliography has emerged since 2020. Some reviews provide thorough descriptions of the current state of the art [2, 3]. Currently, two diagnostic principles dominate the global testing arena: firstly, tests based on the detection of virus-specific nucleotides and, secondly, tests based on the detection of virus-specific antigens and/or virus-specific immunoglobulins [4]. For the first diagnostic principle, reverse transcription polymerase chain reaction (RT-PCR) and loop-mediated isothermal amplification (LAMP) are utilized; while for the second diagnostic principle lateral flow assays (LFA) and/or enzyme-linked immunosorbent assays (ELISA) are utilized. The RT-PCR, detecting the presence of viral nucleotides and typically performed on nasal and/or throat swabs (NTS), are accepted by the World Health Organization (WHO), the Food and Drug Administration (FDA) and other national health organizations as being the ‘gold standard’ [5] for having performance characteristics with sensitivity and specificity scores up to well over 90% [6]. In addition, LFA and ELISA, detecting viral antigens or antiviral immunoglobulins, are also recognized by the WHO, FDA and other national health organizations for the detection of SARS-CoV-2 [7, 8]. The LFA, performed on NTS, is mostly used in point-of-care environments, while the ELISA, performed on serum, is mostly used in hospital environments, with variable sensitivity and specificity scores [7, 8].

For SARS-CoV-2 testing to be meaningful, the concept of *contagiousness* is gaining relevance as a scientific and medical parameter of interest. Public interest is leading to criticism on the effectiveness of the use of the RT-PCR test results as a motivation for mitigating measures that have extreme social and financial consequences for the populations involved [9]. Although consensus on the criteria on how to measure contagiousness has not been reached yet [10], at least two defining parameters can be identified. Firstly, in order to be contagious the causative pathogen needs to be present in a matrix which is (actively) dispersed in the environment by the patient or carrier. Secondly, the causative pathogen needs to be in its replicative (‘active’) state in order to be capable of infecting the recipient after transfer [11]. Although RT-PCR is the diagnostic ‘gold standard’ [5] for the detection of SARS-CoV-2 particles, it is not the gold standard for infectivity [12]. Simply detecting the presence of viral RNA does not comply with the second criterium for contagiousness [12]. Similarly, detecting viral antigens or antibodies directed against it, by LFA or ELISA, also does not comply with the second criterium. Furthermore, the use of serum for measuring viral antigens does not comply with the first criterium for contagiousness. Saliva might replace NTS as preferred testing matrix since it can be obtained less invasively. Saliva could be used to measure active replication of SARS-CoV-2 as it has earned a solid scientific base as a matrix [13, 14]. Importantly, besides the obvious transmission route (droplets/aerosols) of saliva, it may also be transferred from person to person via different mechanisms, including kissing, rendering saliva an ideal vector for a diverse range of pathogens [15].

To detect the infectivity, fluorescence *in situ* hybridization (FISH), an analytical method to detect specific RNA or DNA sequences, can be used. FISH is a rapid and easy method with a high sensitivity and the possibility to detect multiple nucleotides simultaneously. It is a reliable technique that has been optimized in its 20+ years of existence. In addition, by use of specific probes, FISH is able to distinguish between the replicative and non-replicative state of a pathogen [16, 17]. Moreover, FISH has proven to be a valid and practical strategy in the direct *in situ* detection of SARS-CoV-2, utilizing SARS-CoV-2-specific and fluorescently-labeled oligonucleotide probes [18]. In the pilot study presented here, the simultaneous detection of both SARS-CoV-2 sense genomic RNA (viral load) and SARS-CoV-2 antisense genomic RNA (viral replication), by way of FISH in saliva, is tested for its diagnostic potential. In addition, the combination of this method and matrix is used to examine the contagiousness proxy.

## Methods

### Saliva samples

Saliva samples were obtained from 10 healthy persons, 10 mild-COVID-19-symptomatic persons and 10 moderate-COVID-19-symptomatic persons. The saliva used in this pilot study was extracted from the salivary gland ducts in the mouth (whole-mouth saliva), of which 5 ml was collected in a container by the person him/herself. Inclusion criteria for the healthy persons were: persons >18 years of age, no symptoms and no history of SARS-CoV-2 infection. Inclusion criteria for the mild-COVID-19-symptomatic patients were: persons >18 years of age, SARS-CoV-2 RT-PCR positive and symptoms as described in Table 1. Inclusion criteria for the moderate-COVID-19-symptomatic patients were: persons >18 years of age, SARS-CoV-2 RT-PCR positive and symptoms as described in Table 1. Exclusion criteria were a known history of COVID-19 infection and hospitalized persons. All included persons used the commercial services of Biotrack, Leeuwarden to detect the presence of SARS-CoV-2 in their saliva and signed and approved for the use of their saliva sample for anonymous research purposes. Since the saliva samples were anonymous-coded diagnostic rest material, no approval from the Medical Research Ethics Committee was needed, according to the Code of conduct for responsible use (Foundation Federation of Dutch Medical Scientific Societies).

**Table 1:** Symptoms of mild- and moderate-COVID-19-symptomatic patients.

Mild-symptomatic	Moderate-symptomatic
Nasal congestion	Nasal congestion
Runny nose	Runny nose
Sore throat	Sore throat
Low fever	High fever
Dry cough	Deep cough
Fatigue	Fatigue
Headache	Headache
Loss of taste/smell	Loss of taste/smell
Nausea/vomiting	Nausea/vomiting
Diarrhea	Diarrhea
	Chills
	Muscle pain
	Shortness of breath

### Fluorescence *in situ* hybridization (FISH)

Saliva samples underwent a multiplex staining procedure with 4’,6-diamidino-2-phenylindole (DAPI) and 2 fluorescent probes in order to achieve a triple-labeling. DAPI was utilized for general DNA staining. The fluorescent probes, directed at the nucleocapsid (N) gene of SARS-CoV-2, comprised of 1) a Cy3-labeled antisense genomic RNA (agRNA)-specific (+; sense) probe (Biotrack COV19 Probe, 24 nucleotides, GC: 41.7%, Tm: 60.2°C), and 2) its complementary sequence, a FITC-labeled sense genomic RNA (sgRNA)-specific (-; antisense) probe (Biotrack COV19 GProbe, 24 nucleotides, GC: 41.7%, Tm: 60.2°C). Both probes have an *in silico* specificity of 100% and are employed in a serial fashion to circumvent possible duplexing between the two probes. The DAPI stained the general DNA content, the SARS-CoV-2 agRNA probe the replicative form of the virus, and the SARS-CoV-2 sgRNA probe all virus forms (replicative and non-replicative).

To perform the FISH procedure, the saliva was fixed for 10 min. by mixing 5 ml of saliva with 5 ml Biotrack Preservation Fluid. This fixed cell suspension was mixed 1:2 (50 μl: 100 μl) with the Biotrack COV19 Probe, whereafter the mixture was hybridized at 50°C for 1.5 hour. Thereafter, 1 ml Biotrack Wash Buffer was added for 30 min. at 60°C. Subsequently, the suspension was centrifuged (10 min. at 800 x g), the supernatant removed, and the pellet resuspended with 100 μl Biotrack COV19 GProbe, whereafter the mixture was hybridized at 50°C for 1.5 hour. Again, 1 ml Biotrack Wash Buffer was added for 30 min. at 60°C, after which the suspension was centrifuged (10 min. at 800 x g), the supernatant removed, and the pellet resuspended with 100 μl DAPI-mix, whereafter the mixture was hybridized at 50°C for 15 min. Next, 1 ml Biotrack Wash Buffer was added and the triple-labeled suspension was washed for 30 min. at 60°C. Finally, the suspension was centrifuged (10 min. at 800 x g), the supernatant removed, and the pellet resuspended with 50 μl Milli-Q. From every saliva sample (n = 30), 10 μl was spotted onto a microscopic slide.

In addition, a positive and negative analytical control were analyzed according to the FISH procedure described above (data not shown) to ensure that the procedure was performed correctly.

### Data acquisition and interpretation

For microscopic data acquisition, a Leica DM2500 fluorescence microscope equipped with a Leica EL6000 mercury lamp and a Leica DFC450C camera was used. Images were taken with the software Leica application suite V4.20 and stored in JPG-format.

To study the cellular content of the saliva samples [19], images with DAPI and bright field were taken. A magnification of 400x was used to photograph the samples. For every image, the gain was set to 1.0 and the exposure time was set at 150 ms for DAPI and bright field. These images where thereafter examined for the presence of epithelial cells and white blood cells, based on size and morphology.

The FISH triple-labeling was visualized by a magnification of 400x or 1000x. For every image, the gain was set to 1.0 and the exposure time was set at 150 ms for DAPI (and bright field) and at 500 ms for Cy3 and FITC.

### Biotrack-MED analysis

The Biotrack-MED® analyzer (patent EP 08874964.3, CE-IVD: NL-CA002-2020-51055) is a semi-autonomous, multi-sample filter cytometer (Biotrack, Leeuwarden, The Netherlands) [20]. The multi-layer neural network of the analyzer was trained for SARS-CoV-2 interpretation with the images acquired above. After training of the multi-layer neural network of the analyzer, 30 μl of each of the triple-labeled saliva samples was spotted on a plastic sample container (filter cup). The filter cups were fitted into an analysis disk and placed into the Biotrack-MED® analyzer with the SARS-CoV-2 application. The network produced per sample a microscopic fluorescence 3D image (magnification 200x, approximately 150 images per sample), in which objects were isolated by computer vision algorithms. Subsequently, these computer vision algorithms calculated morphometric parameters (n = 20, including shape, area, roundness, brightness and number of spots) per object. With this information, the multi-layer network was then able to interpret the microscopic image as being *positive* or *negative* for SARS-CoV-2. The analyzer is able to perform the above in an automated manner and can be set up to analyze up to 200 samples (n = 18 per run) in one workday.

## Results

### Suitability of saliva as matrix for SARS-CoV-2 detection

**Fig 1:**
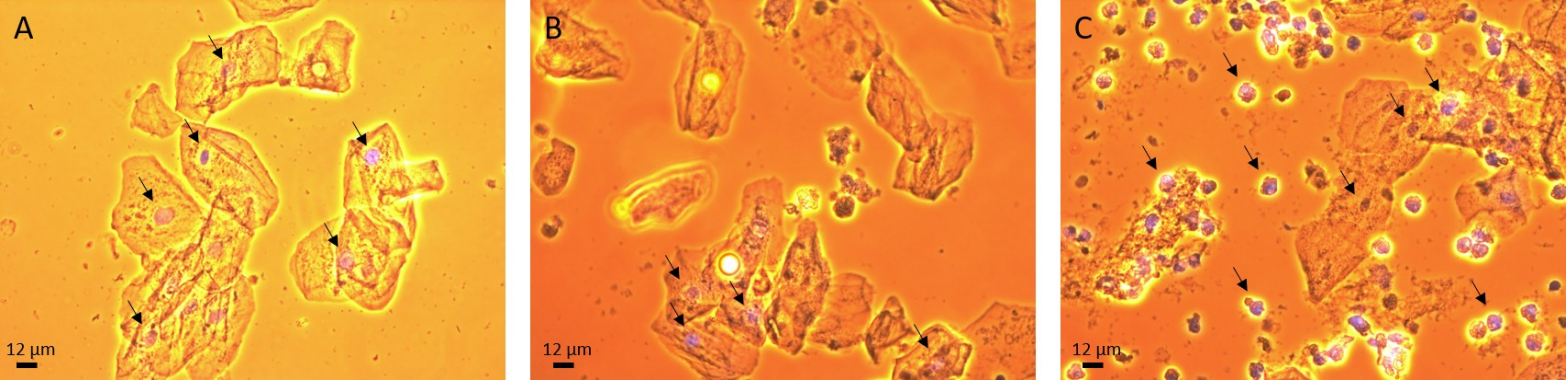
Cellular content in saliva samples. The saliva samples were stained with DAPI (blue, arrows) to fluorescently label the cell nuclei of **A**) a healthy person, **B**) a person with mild symptoms and **C**) a person with moderate symptoms.

To determine whether saliva is a suitable matrix for SARS-CoV-2 testing, the cellular content of the saliva samples was studied after staining the general DNA content with DAPI. The cellular content consisted of epithelial cells and white blood cells in healthy, mild-symptomatic and moderate-symptomatic persons (Fig 1). Interestingly, the moderate-symptomatic persons showed a higher number of white blood cells (Fig 1C), compared to the healthy and mild-symptomatic persons. Thus, the cellular content of saliva was suitable for SARS-CoV-2 examination.

### Microscopic evaluation of viral load and viral replication in saliva samples

To test the diagnostic potential, saliva samples were triple-labeled with DAPI and the agRNA and sgRNA probes for SARS-CoV-2. With this triple-labeled staining, information about the general DNA content (DAPI), viral replication (SARS-CoV-2 agRNA probe, detecting the ‘active’, replicative form) and viral load (SARS-CoV-2 sgRNA probe, detecting the ‘presence’ of both replicative and non-replicative forms).

**Fig 2:**
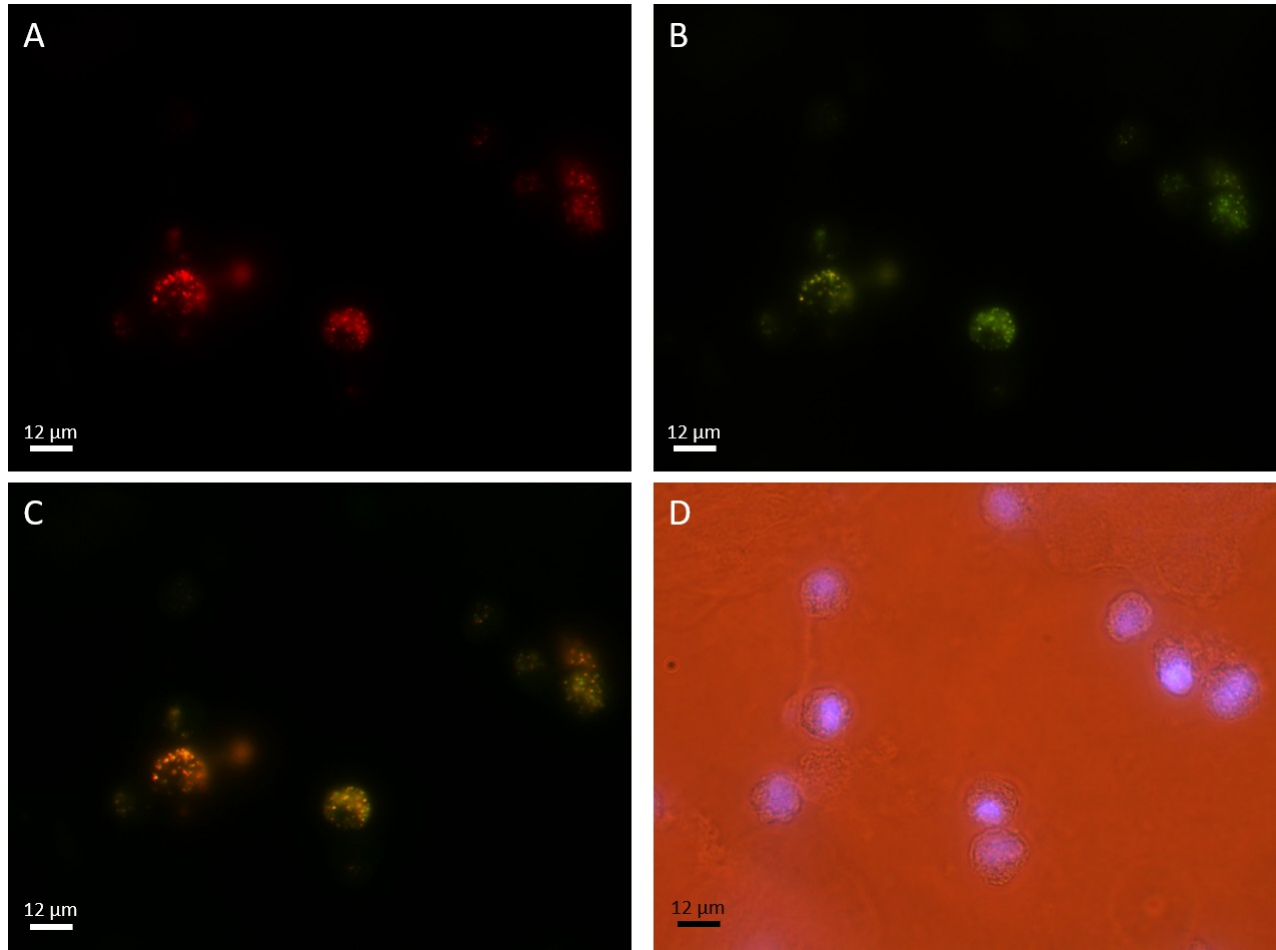
Triple-labeling of a saliva sample. The saliva sample was stained for **A**) SARS-CoV-2 antisense genomic RNA (Cy3, red), **B**) SARS-CoV-2 sense genomic RNA (FITC, green). A combination of both SARS-CoV-2 antisense and sense genomic RNA is depicted in **C** and nuclei are visualized in **D** (DAPI, blue). The viral particles depicted in **A** are in the replicative form, while **B** depicts all viral particles present, both in the replicative and non-replicative forms.

**Fig 3:**
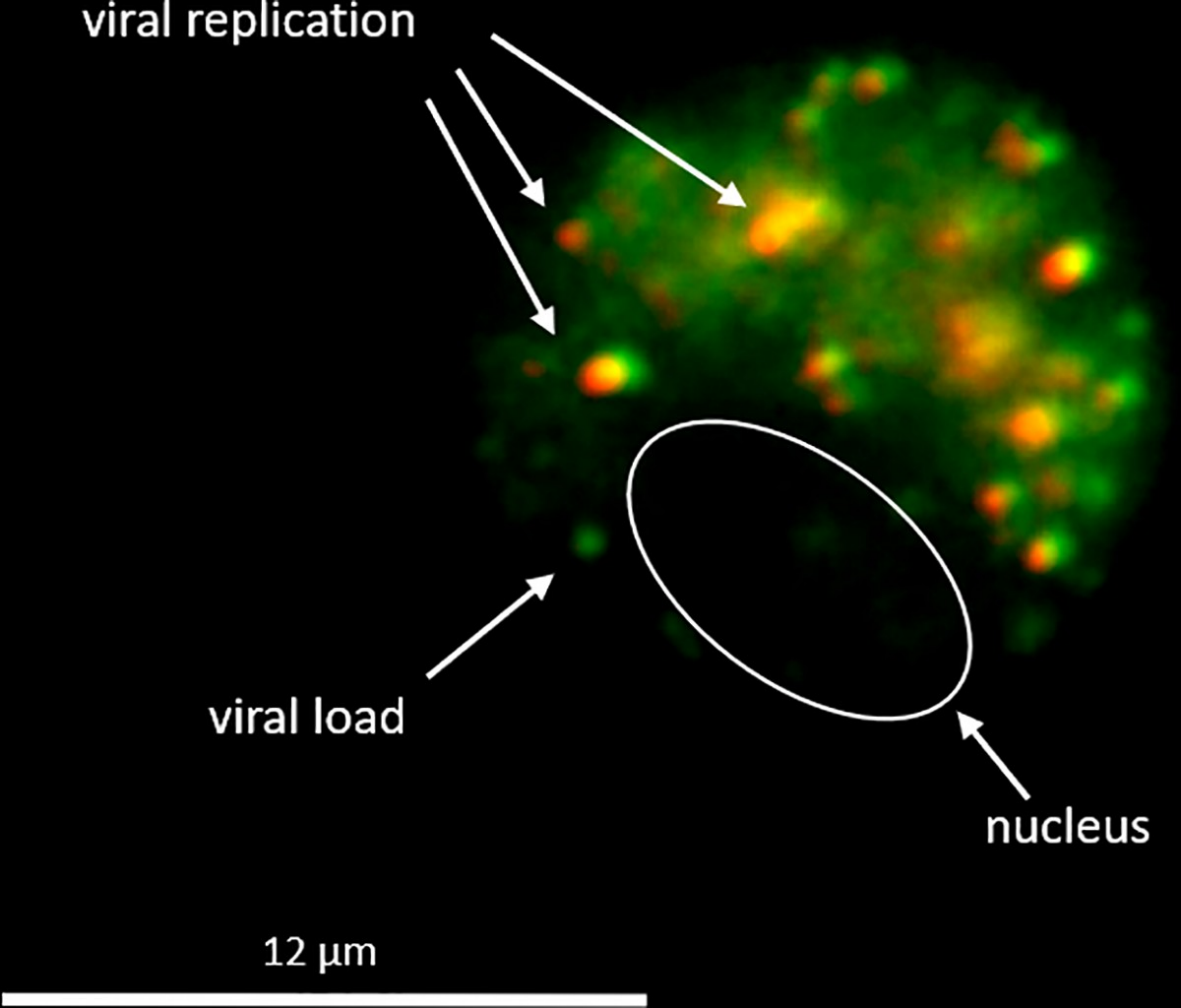
Triple-labeling show viral replication. SARS-CoV-2 sense genomic RNA (green) indicates viral nucleotides (all viral particles, both replicative and non-replicative), while SARS-CoV-2 antisense genomic RNA (red) indicates viral nucleocapsid proteins (replicative viral particles). The viral load of the cell is indicated by the green color, while viral replication is indicated by the yellow-red color.

Triple-labeling yielded microscopic images that detected SARS-CoV-2 in saliva (Fig 2), and distinguished between the replicative and non-replicative forms of this virus (Fig 3).

### Saliva as a possible contagiousness proxy

**Fig 4:**
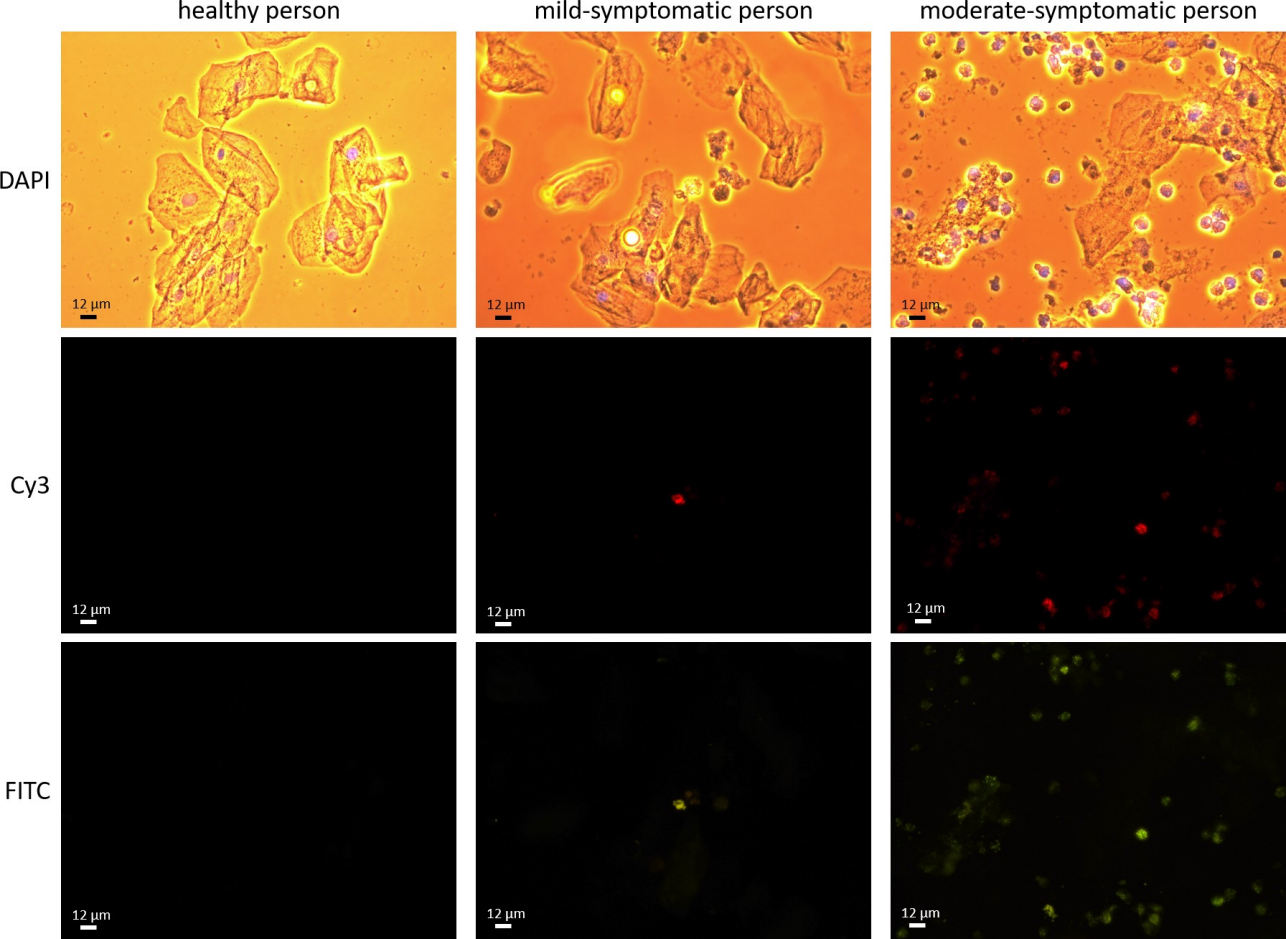
Triple-labeling of saliva samples of a healthy, a mild-symptomatic, and a moderate-symptomatic person. The saliva samples were stained for DAPI (blue), SARS-CoV-2 antisense genomic RNA (Cy3, red) and SARS-CoV-2 sense genomic RNA (FITC, green). The Cy3 depicts the replicative form of SARS-CoV-2, while the FITC depicts both the replicative and non-replicative form of SARS-CoV-2.

Next, the possible qualitative properties of this method were tested. The healthy persons showed no presence of either replicative (agRNA) or non-replicative (sgRNA) SARS-CoV-2, while the mild-symptomatic persons had a small amount of white blood cells with evidence of presence of both agRNA and sgRNA of SARS-CoV-2 (Fig 4). The moderate-symptomatic persons showed in almost all white blood cells the presence of both agRNA and sgRNA of SARS-CoV-2 (Fig 4). Thus, the severity of the symptoms may be an indication of the amount of replicative virus present in saliva.

### Automated detection of SARS-CoV-2 agRNA by the Biotrack-MED analyzer

The saliva samples of the 30 included persons were tested with the SARS-CoV-2 application of the Biotrack-MED® analyzer. The SARS-CoV-2 application was obtained by training the multi-layer neural network of the analyzer with similar images as presented in Fig 4. The results of the auto-analyses are presented in Table 2 and show a logic relation with the symptoms. Thus, the Biotrack-MED® analyzer and its SARS-CoV-2 application appeared to be capable of detecting replicative SARS-CoV-2 in this pilot study.

**Table 2:** Results of the SARS-CoV-2 application of the Biotrack-MED® analyzer.

Person	Symptoms	SARS-CoV-2 (Biotrack-MED)
1–10	No	10 negative
11–20	Mild	9 positive, 1 negative
21–30	Moderate	10 positive

## Discussion

In this pilot study, we examined the diagnostic potential of the simultaneous detection of SARS-CoV-2 sense genomic RNA (viral load) and SARS-CoV-2 antisense genomic RNA (viral replication) in saliva. In addition, the contagiousness proxy was examined. Our results show that saliva is a suitable matrix for detection of SARS-CoV-2, utilizing fluorescent *in situ* hybridization. Both viral load and viral replication can be detected in saliva and the abundance of the replicative form may give information about the contagiousness of the person. Finally, we demonstrated that viral replication can be detected in an automated manner by the SARS-CoV-2 application of the Biotrack-MED® analyzer.

Fluorescent *in situ* hybridization, using sense and antisense genomic RNA probes directed against specific regions of the N gene of SARS-CoV-2, generated interpretable fluorescent images, with the sense genomic RNA probe indicative for viral load and the antisense genomic RNA probe indicative for viral replication. FISH is a reliable technique used more than 20 years with a high sensitivity. The detection threshold levels are as low as 10–20 copies of RNA per cell [21, 22]. The amount of the antisense genomic RNA staining (Cy3, replicative form) in relation to the sense genomic RNA staining (FITC, replicative and non-replicative form), as shown in Fig 4, may be indicative for a dose-response relationship between the number of positively labeled (white blood) cells and disease stage severity. This might give an indication about potential contagiousness [23]. Thus, the triple-labeling of saliva meets the two parameters of contagiousness measurement: SARS-CoV-2 is present in saliva, a matrix that is dispersed in the environment, and SARS-CoV-2 is present in its replicative state.

The use of saliva as a matrix for SARS-CoV-2 detection has gotten increased attention the last few months. One of the main advantages of utilizing saliva for SARS-CoV-2 testing is that it is a non-invasive method for which no trained personnel is necessary. It is an easily accessible matrix, compared to blood or NTS, and both viral RNA and antibodies are present in saliva [24, 25]. As stated before, saliva is a matrix that is easily dispersed in the environment, thereby a potentially source of contamination. In line with this, a study of Smither et al. showed that experimental aerosols with SARS-CoV-2 were able to survive as long as 90 minutes [26]. Moreover, SARS-CoV-2 infections are present in the cells of the salivary glands, which resulted in the transmission of SARS-CoV-2 in the environment [27]. It is no surprise that SARS-CoV-2 is present in and can be transmitted through saliva. Over the last years, several viruses could be detected in saliva, including, including Epstein-Barr Virus, cytomegalovirus, norovirus, influenza A/B virus, rabies virus, hepatitis B/C virus and herpes simplex virus [28].

One observation of this pilot study was the high amount of white blood cells in the persons with moderate COVID-19-associated symptoms. In the field, there is no consensus about this topic, as some studies show increased [29], while other studies show decreased [30] amount of white blood cells in COVID-19 patients. However, these studies have been conducted in blood, and not saliva. It is known that white blood cells in saliva are increased upon pathogen invasion [31], which may also be the case in SARS-CoV-2 infected persons.

As far as we know, a SARS-CoV-2 test as examined in this pilot study has not been published before. The test described in this pilot study is able to generate simultaneously information on the presence of sense genomic RNA–indicative of the presence of the pathogen–and on the presence of antisense genomic RNA–indicative of activity of the pathogen. In this view, our test might have the potential to be superior to the RT-PCR, the diagnostic ‘gold standard’ [5] for SARS-CoV-2 at this moment, as our test does not only examine the presence of the virus, but also detect whether the virus is still replicating and, thus, is potentially infective. The RT-PCR only detects the presence of viral RNA, but cannot make a distinction between ‘replicating’ or ‘non-replicating’ viral particles [12]. While the contagiousness of SARS-CoV-2 is less than 10 days, viral RNA can be detected by RT-PCR for several weeks [32, 33]. At the moment, the contagiousness can only be determined by viral cell culture. This may take up to 4 days, or even more [34] and requires a BSL3 certified laboratory. The method described in this pilot study is able to determine within 24 hours whether a person is contagious or not. Besides the increased speed of the test, there is no need for a BSL3 laboratory to perform the test since the collection medium inactivates the sample within 10 minutes.

This small pilot study shows that both the viral particles and the intracellular location where the viral N gene is expressed are solely observed intracellularly and mostly at identical locations. Given the phagocytic and immunological function of white blood cells, it is not surprising that these cells show a significantly higher presence of SARS-CoV-2 compared to epithelial cells. Both probes utilized in this pilot study yield sharp-edged, high intensity spots, which renders the obtained images especially suitable for automated image analysis and subsequent use of a neural network for interpretation. Consequently, the combination of a simplified protocol with an automated, multi-sample analyzer system allows for the rational application of FISH-based methods in high-throughput scenarios.

A limitation of this pilot study is the small number of inclusions. A larger study is needed to be able to draw a firmer conclusion and provide sensitivity and specificity studies, although a challenge will be the determination of the true ‘gold standard’ due to the broad range of these scores for the RT-PCR [6] and the lack of studies on true infectivity potential [12]. In regards to the latter, viral replication and infectivity can be confirmed by cell culture. However, as SARS-CoV-2 is classified as a risk group 3 biological agent by the European Commission, the laboratory available for this study was not permitted to perform these experiments.

In conclusion, this pilot study shows that both sense and antisense genomic RNA probes specifically targeting the N gene of SARS-CoV-2 are suited for *in situ* applications in human saliva. Moreover, the diagnostic strategy of *in situ* and qualitative measurement of SARS-CoV-2 activity in saliva, indicated by the antisense genomic RNA expression, may be a proxy of contagiousness.
